# Evaluating smartphone strategies for reliability, reproducibility, and quality of VIA for cervical cancer screening in the Shiselweni region of Eswatini: A cohort study

**DOI:** 10.1371/journal.pmed.1003378

**Published:** 2020-11-19

**Authors:** Ramin Asgary, Nelly Staderini, Simangele Mthethwa-Hleta, Paola Andrea Lopez Saavedra, Linda Garcia Abrego, Barbara Rusch, Tombo Marie Luce, Lorraine Rusike Pasipamire, Mgcineni Ndlangamandla, Elena Beideck, Bernhard Kerschberger

**Affiliations:** 1 Medecins Sans Frontieres, Geneva, Switzerland; 2 George Washington University, Washington, District of Columbia, United States of America; 3 Weill Cornell Medical College, New York, New York, United States of America; 4 Eswatini Ministry of Health, Mbabani, Eswatini; Harvard University, UNITED STATES

## Abstract

**Background:**

Cervical cancer is among the most common preventable cancers with the highest morbidity and mortality. The World Health Organization (WHO) recommends visual inspection of the cervix with acetic acid (VIA) as cervical cancer screening strategy in resource-poor settings. However, there are barriers to the sustainability of VIA programs including declining providers’ VIA competence without mentorship and quality assurances and challenges of integration into primary healthcare. This study seeks to evaluate the impact of smartphone-based strategies in improving reliability, reproducibility, and quality of VIA in humanitarian settings.

**Methods and findings:**

We implemented smartphone-based VIA that included standard VIA training, adapted refresher, and 6-month mHealth mentorship, sequentially, in the rural Shiselweni region of Eswatini. A remote expert reviewer provided diagnostic and management feedback on patients’ cervical images, which were reviewed weekly by nurses. Program’s outcomes, VIA image agreement rates, and Kappa statistic were compared before, during, and after training. From September 1, 2016 to December 31, 2018, 4,247 patients underwent screening; 247 were reviewed weekly by a VIA diagnostic expert. Of the 247, 128 (49%) were HIV–positive; mean age was 30.80 years (standard deviation [SD]: 7.74 years). Initial VIA positivity of 16% (436/2,637) after standard training gradually increased to 25.1% (293/1,168), dropped to an average of 9.7% (143/1,469) with a lowest of 7% (20/284) after refresher in 2017 (*p* = 0.001), increased again to an average of 9.6% (240/2,488) with a highest of 17% (17/100) before the start of mentorship, and dropped to an average of 8.3% (134/1,610) in 2018 with an average of 6.3% (37/591) after the start of mentorship (*p* = 0.019). Overall, 88% were eligible for and 68% received cryotherapy the same day: 10 cases were clinically suspicious for cancer; however, only 5 of those cases were confirmed using punch biopsy. Agreement rates with the expert reviewer for positive and negative cases were 100% (95% confidence interval [CI]: 79.4% to 100%) and 95.7% (95% CI: 92.2% to 97.9%), respectively, with negative predictive value (NPV) (100%), positive predictive value (PPV) (63.5%), and area under the curve of receiver operating characteristics (AUC ROC) (0.978). Kappa statistic was 0.74 (95% CI; 0.58 to 0.89); 0.64 and 0.79 at 3 and 6 months, respectively. In logistic regression, HIV and age were associated with VIA positivity (adjusted Odds Ratio [aOR]: 3.53, 95% CI: 1.10 to 11.29; *p* = 0.033 and aOR: 1.06, 95% CI: 1.0004 to 1.13; *p* = 0.048, respectively). We were unable to incorporate a control arm due to logistical constraints in routine humanitarian settings.

**Conclusions:**

Our findings suggest that smartphone mentorship provided experiential learning to improve nurses’ competencies and VIA reliability and reproducibility, reduced false positive, and introduced peer-to-peer education and quality control services. Local collaboration; extending services to remote populations; decreasing unnecessary burden to screened women, providers, and tertiary centers; and capacity building through low-tech high-yield screening are promising strategies for scale-up of VIA programs.

## Abstract 2

Ramin Asgary and colleagues evaluate a program for improving digital cervicography via telemedicine to serve women in remote settings.

## Author summary

**Why was this study done?:**

- Visual inspection of the cervix with acetic acid (VIA)-based cervical cancer screening is the World Health Organization (WHO)-recommended screening method for resource-poor settings, and new advanced human papillomavirus (HPV) testing also requires a reliable, good-quality, and functioning VIA or cytology program.
- Initial VIA training programs for cervical cancer screening are often short term with variable quality, and opportunities for retraining are limited due to cost and logistics, which contribute to a decline in diagnostic skills of screening providers with high false-positive and negative results over time.
- Cervical imaging, cervicography, as a proven adjunct to VIA, improves screening results and competencies of providers but requires a digital camera and television screen, which poses scalability and sustainability issues.
- Smartphone-based VIA strategies provide a low-tech option to improve training of nurses in VIA, but their large-scale implementation and the impact on quality in routine program settings have not yet been evaluated.

**What did the researchers do and find?:**

- We developed and implemented an integrated mentorship training of VIA screening and digital cervicography using smartphones through a telemedicine platform to improve provider nurses’ diagnostic and management competencies and skill retention to ensure reproducibility, reliability, and quality of VIA screening services in routine humanitarian settings.
- Our findings indicate that provider nurses significantly improved in VIA performance and maintained diagnostic competencies with high agreement rates with the expert mentor over time after smartphone-based mentorship training compared to the standard and refresher VIA screening trainings.
- In resource-poor settings, these achievements could help reduce the unnecessary burden of underdiagnosis or overdiagnosis and treatment and their psychosocial impact to women, providers, and health systems.

**What do these findings mean?:**

- The strategy of using inexpensive and ubiquitous smartphones presents a unique opportunity for task shifting of high-quality cervical cancer screening at the primary care level in many resource-poor or humanitarian settings.
- Effective collaborations with local entities, local capacity building, maintenance of quality services, decreased burden on health systems, and direct affordable VIA screening services for women in remote areas and in familiar and sustainable settings could lead to important policy implications for VIA screening interventions in humanitarian settings.

## Introduction

Worldwide, more than half a million women develop cervical cancer every year, and more than half of these cases are fatal [1]. In resource-poor settings, cervical cancer is the leading cause of cancer-related deaths among women. This is mainly related to inadequate screening and HIV infection [2,3]. Extensive study of visual inspection of the cervix with acetic acid (VIA) indicates acceptable test characteristics and performance, [4–7] comparable with Pap testing, which is unavailable in most developing regions [8]. However, despite the availability of VIA, the screening rate is unacceptably low in many low-resource or humanitarian settings [9–11]. Barriers to cervical cancer screening include lack of a nonphysician health workforce and infrastructure for follow-up [9,12].

Task shifting by training nurses in VIA has been effective in low-resource settings [13–18], but there are major barriers including lack of comprehensive training of nonphysician health workers and opportunities for repeated training [18–21]. This contributes to a decline in diagnostic and management accuracy over time [19,20]. Cervicography, a well-proven adjunct to VIA, allows mid-level health professionals to review and discuss cervical images with mentors to assure long-term screening accuracy [13,14,18]. However, it requires a digital camera and television screen [13,14,18], which poses scalability and sustainability issues. Smartphone cameras provide a reliable alternative option. Although some pilot research projects have tested this approach [15,16,22], it has not yet been evaluated in routine settings.

The incidence of cervical cancer in Eswatini, at 69.4 to 75.3 per 100,000, is among the highest in the world [11,23]. Eswatini’s first national policy on cervical cancer prevention, developed in 2013, recommended VIA screening with cryotherapy of precancerous lesions for women aged 25 to 49 years. However, most women in Eswatini have never been screened [11]. The rural Shiselweni region in Eswatini has a 31% HIV prevalence (ages 18 to 49 years), which likely increases the risk for early and accelerated progression of disease and greater severity at presentation. We developed and implemented an integrated mentorship training of VIA/digital cervicography via telemedicine using smartphones to improve nurses’ diagnostic and management competencies and skill retention. This paper aims to describe the development, implementation, and assessment of this training program and its effect on reproducibility, reliability, and program-level VIA quality.

## Methods

### Setting, study sites, and participants

Beginning in September 2016, in collaboration with the Ministry of Health (MoH) of Eswatini, Medecins Sans Frontieres (MSF) implemented a VIA screening program under routine conditions for women aged 25 to 49 years in primary and secondary facilities in the rural Shiselweni region, with a population of 204,000. This region is among the most remote and underserved areas in Eswatini, where there is limited access to healthcare, especially for women, high rates of HIV, and relatively inaccessible terrain with large distances between health facilities. Nurses and midwives (*n* = 3; 2 female) were recruited, trained, implemented VIA, provided cryotherapy for precancerous lesions, and referred advanced or not cryotherapy-eligible cases. MSF provided supplies and logistical support to perform VIA, cryotherapy, and treatment of infections detected during vaginal exams free of charge.

Nurses performed VIA in HIV/tuberculosis (TB) care integrated outpatient primary (*n* = 9) and secondary care facilities (*n* = 1) and performed VIAs on all eligible women (25 to 49 years of age) between September 2016 and December 2018. All women <25 years or >49 years who requested were also screened with VIA or Pap testing, respectively, following national protocol. For sustainability and scalability of the intervention’s effect, toward the end of the project and upon handover to MoH in 2019, the nurses trained in this program were recognized as trainers and continued to provide mentorship to MoH nurses in a total of 30 facilities. Pelvic exams and VIA screenings took place in MoH facilities where patients provided usual consent to care. Patients diagnosed with precancerous lesions were offered same-day or follow-up cryotherapy by nurses in the same facility. Patients who did not meet criteria for cryotherapy, which included lesions covering more than 75% of the cervical surface and/or lesions extending beyond 2 mm inside the os or to the vaginal wall, were referred to the central regional hospital. In accordance with MoH’s plan, all cancer cases were referred to national gynecologists or included in a waiting list for transfer of care to a dedicated hospital in South Africa for radiotherapy or surgery. All VIA and subsequent care were provided free of charge. As part of program evaluation, team meetings were frequently held to discuss challenges and elicit feedback from VIA providers, MoH managers, local and national health policy makers, patients, and other stakeholders such as community informants and leaders. We incorporated feedback in our ongoing program evaluation to adjust to logistical, sociocultural, and systems challenges. At the end of the intervention, based upon an original agreement with the MoH, a detailed instruction manual of the mHealth mentorship was developed for the MoH for possible replication of the program.

This research was exempted from the MSF Ethics Review Board for a full review and was approved as a posteriori analysis of routinely collected clinical data by MSF Switzerland Medical Department and approved by the MoH and the Social Welfare Scientific Ethics Committee of Eswatini. Cervical images were obtained after verbal consent and submitted for review without any identifiable information or protected health information (PHI). These images were not recorded in patients’ file as there were no such capabilities in the health facilities. All patients who were seen and cared for during the program implementation as part of services provided by MSF and in the health facilities supported by Eswatini’s MoH provided verbal consent to usual care and that their cervical images will be obtained for better diagnostic of their condition and improving their medical care, quality control purposes and monitoring and evaluation, and for teaching of other providers. This study is reported as per the Strengthening the Reporting of Observational Studies in Epidemiology (STROBE) guideline (S1 STROBE Checklist).

### Description of program and training curriculum

All nurses participated in an initial 1-week on-site training and received a refresher after 1 year. We then introduced a 6-month smartphone-based cervicography mentorship. The hypothesis was that remote mentorship using smartphone-based imaging with feedback from an expert mentor would improve the quality of screening, reliability and reproducibility of VIA diagnostic and management abilities of nurses, treatment of positive cases, and reduce false-positive and negative cases. This, in return, would reduce the unnecessary diagnosis and treatment of false-positive women, burden on treating providers, psychological impact of false precancerous diagnosis, and patient-level burden of false-negative diagnosis.

### Curriculum structure

The VIA training included 2 distinct components: (1) a 1-week initial standard VIA training, which was offered by Eswatini’s MoH, modeled after the standard internationally recognized Johns Hopkins Program for International Education in Gynecology and Obstetrics (JHPIEGO) [24] and included didactic and practical sessions; and (2) a mentorship plan that included 2 sections: (a) an adapted refresher training; followed by (b) 6 months of smartphone-based remote mentorship via telemedicine. The telemedicine platform is a secure closed loop system operated directly by MSF that connects clinical experts and specialists directly to providers in the field.

- The on-site refresher training was offered in Shiselweni in July 2017. The expert/mentor first observed all nurses in their clinics, assessed competencies in GYN exams and VIA procedure, cervical cancer screening counseling, infection control procedures, and treatment and follow-up processes. The mentor obtained cervical images during these VIA exams and provided practical training for smartphone imaging of the cervix. These observations informed the development of a refresher module focused around practice gaps and challenges. A PowerPoint presentation was developed that included a description of each case, cervical images before and after acetic acid application and after cryotherapy, review of diagnostic features and diagnoses made by the nurses, rationale for final diagnoses, and therapeutic approaches. First, a 4-session classroom training included review of female genital anatomy, discussion of human papillomavirus (HPV), cervical cancer, characteristics of cervical cancer in HIV–positive patients, an approach to common mistakes or VIA diagnostic challenges, effective cancer screening, population- and individual-level considerations, the World Health Organization (WHO) screening recommendations, treatment approaches, and cervical photography. Second, during 4 additional sessions, the PowerPoint was reviewed along with a review of technical difficulties in VIA performance. Slides and flash cards from JHPEIGO were also reviewed. One expert reviewer/mentor performed all trainings. WHO, JHPIEGO, additional articles, and personal database were used to develop this training [2,24].
- Around 1 year after the refresher training, due to a rising positivity rate, a targeted strategy was implemented. Different smartphone cameras were tested, and Samsung Duo was selected and purchased locally specifically for screening purposes. All nurses were trained in cervical photography and given written instructions. SIM cards were removed from the phones, which were password protected. The smartphones were used only for their capability to take digital photos through the camera application, and no specific applications for transmission of the images was used. The smartphones were collected at the end of the day and were connected to a password-protected laptop. Then, a secure, online telemedicine-supported platform was used to transfer images and diagnoses to the expert reviewer and back to the nurses. This platform was introduced for a 6-month period during which nurses would (a) capture cervical images before and after acetic acid application; (b) record patients’ demographic, clinical, and contact information; (c) document diagnoses and management recommendations; and (d) de-identify, codify, and submit images to the reviewer who was overseas (S1 CaseTemplate). Nurses uploaded images daily from their password-protected phones to a password-protected computer. One nurse then submitted the images weekly to the reviewer. Still, VIA images were captured/transferred in JPEG format, 3024 × 4032.

Based on Adult Learning Theory and in a trainee-centered approach, nurses had the liberty to prioritize challenging positive and negative cases first. They were also required to select a random sample of 1 to 2 cases from each day in the clinic depending on the number of patients seen (1 case if less than 10 patients seen, 2 cases if more than 10 patients seen). Only 1 expert reviewer/mentor, a cancer prevention specialist with extensive experience in VIA diagnostic and performance, training, implementation, and assessment in low-resource settings, reviewed all images, hence no interobserver variability. The expert reviewer reviewed images weekly; provided diagnoses, management recommendations, and rationale; annotated images for clarification; and submitted the results to the field team. Since the primary purpose of image review was to provide tailored mentorship and training, observe individual’s weakness and strengths, and provide individual level feedback and education modeled after Adult Learning Theory, the expert reviewer was not blind to nurses’ diagnosis; however, images were first reviewed, and then the provider identity was observed. The field team convened weekly or biweekly to review and discuss all cases. They also shared lessons regarding improving cervical photography and other logistical barriers.

Nurses were trained in quality photography, and a cheat sheet was developed to help with better techniques in quality photography (S1 Text). The expert trainer/reviewer also provided regular support and feedback to address potential quality issues with cervical images.

### Study design, evaluation, and analysis plan

In this single-arm experimental cohort study, nurses documented patients’ demographics and VIA and cryotherapy outcomes using the routine national cervical cancer registry for all patients seen for VIA. The evaluation included an assessment of training efficacy and a retrospective cohort analysis of VIA diagnoses of women aged 25 to 49 years. The primary outcomes included agreement rates of VIA image interpretation between the nurses and the expert reviewer during the 6-month mentorship as a subsample of the total patient population receiving VIA. The secondary outcomes included VIA positivity and cancer suspect rates at the program level, which were compared before, during, and after the refresher training and before, during, and after the 6-month mentorship. “Cancer suspect” refers to cases that were clinically suspicious for cancer but not yet confirmed with biopsy. The expected positivity rate of cervical precancerous lesions in the adult female population of Eswatini during the implementation period was also estimated. Additionally, stakeholders’ input and feedback during the implementation phase were explored.

The categories of diagnostic interpretation were defined as (1) VIA/cervicography–negative—no atypical acetowhite changes in the transformation zone; (2) VIA–positive—lesions need cryotherapy or referral for further intervention; (3) suspicious for cancer—need biopsy for confirmation. In addition to simple agreement rates, and to account for random error, Cohen Kappa statistic was used to measure the reproducibility of screening test diagnoses, which was considered none to slight, fair, moderate, substantial, and almost perfect for ≤0.20, 0.21 to 0.40, 0.41 to 0.60, 0.60 to 0.80, and 0.80 to 1.00, respectively. Statistical significance was defined as *p* < 0.05. Frequency statistics, proportions, medians, means and interquartile ranges were used to describe percentage of patients needing and receiving cryotherapy, number of cancer suspects, and baseline characteristics of the screened population, when available. Bivariate analyses with 1 predictor at a time as well as multivariable logistic regression analyses with age and HIV status as predictors in the model were used to better characterize sociodemographic and patient-level indicators in relation to VIA outcomes. The changes in the rates of the VIA outcomes were compared over the study period using linear regression models as there are no sufficient data to suggest that the relationship between VIA outcomes and age differs in different age categories of women eligible for VIA. The odds ratios (ORs) for VIA positivity were measured by comparing the number of VIA–positive cases between different timeframes from the pre-refresher to post-mentorship periods. STATA (StataCorp, V 14, College Station, Texas, United States of America) was used for data analysis.

To estimate the expected/ideal rate of VIA positivity and precancerous lesions in Eswatini, we considered multiple factors, each with some level of uncertainty due to data scarcity, including the cervical cancer incidence estimation in Eswatini (70 per 100,000) [11,23], the progression rate of precancerous lesions (40% over 5 to 10 years, depending on HIV status), the natural history of cervical cancer (4 years on average), and the number of Eswatini’s national cervical cancer diagnoses. We concluded that the precancerous VIA–positive lesions and cancer suspect cases should roughly be 2% to 4%.

## Results

### Training evaluation

From September 1, 2016 to December 31, 2018, 4,247 patients underwent screening. Overall, images from 247 of both negative and positive cases (6% of total VIA screened patients) were reviewed by the expert reviewer. Twenty-four of all submitted images were of low quality, which were not included in 247 cases.

Table 1 presents sociodemographic of women who underwent VIA. Table 2 presents sociodemographic and clinical indicators of women who underwent smartphone-based VIA. Table 3 presents agreement rates data for all nurses, collectively. There were no significant differences between average agreement rates for the first 3-month and 6-month periods. However, a Kappa of 0.64 (substantial) after the first 3 months improved to 0.79 at the end of the 6-month period. A very high average negative predictive value (NPV) and a modest positive predictive value (PPV) were achieved. Area under the curve of receiver operating characteristics (AUC ROC) was estimated at 0.97.

**Table 1:** Sociodemographic of women screened by VIA, Shiselweni, Eswatini, September 2016 to February 2018.

		Total Number (%)	Number of VIA Negative (row %)	Number of VIA Positive (row %)	p-value
**Age group**	<25	125 (11.8)	95 (77.2)	28 (22.8)	0.40
	25-49	542 (51.3)	401 (75.7)	129 (24.3)	
	>49	41 (3.9)	34 (85)	6 (15)	
	Missing	349 (33.0)	327 (94.0)	21 (6.0)	0.001
	Mean (SD)	33.9 (9.4)	34.1 (9.79)	33 (8.08)	0.32
**Parity**^**1**^	Nulliparous	53 (5)	47 (88.7)	6 (11.3)	0.65
	1	187 (17.8)	153 (82.3)	33 (17.7)	
	2	254 (24.1)	207 (82.1)	45 (17.9)	
	3+	559 (53.1)	446 (81.7)	100 (18.3)	
	Mean (SD)	3.04 (2.1)	3.04 (2.14)	2.96 (1.88)	0.67
**HIV status**^**2**^	Positive	500 (47.9)	406 (82.5)	86 (17.5)	0.86
	Negative	544 (52.1)	440 (82.1)	96 (17.9)	
**ART (among 500 HIV+)**	On ART	473 (95)	381 (81.9)	84 (18.1)	0.18
	Not on ART	26 (5)	24 (92.2)	2 (7.7)	
**Screening status**	First	968 (91.8)	772 (81.1)	180 (18.9)	0.001
	Second	97 (8.3)	83 (95.4)	4 (4.6)	

* Total number 1,057 with 1,041 known VIA status.

** 16 patients with VIA result unknown or missing^1^; Parity is unknown for n = 4 ^2^; 13 patients with HIV status unknown (11 VIA negative, 2 VIA positive); 1 patient with unknown ART status

***Due to changes in cervical cancer registry from Ministry of Health and other logistical challenges some sociodemographic data of the patients were not recorded consistently at the program level and outside reviewed cases.

**Table 2:** Sociodemographic and clinical indicators of women undergoing smartphone VIA, Shiselweni, Eswatini, 2016–2018.

	Mean (SD)		Number (%)
**Age**	30·80 years (SD:7·74 years)	**HIV status**	128/247 (49%)
**Parity**	2.56 (SD: 1.72)	**History of STIs**	8/247 (3.2%)
		**Previous VIA**	25/247 (10%)

**Table 3:** Nurses’ training evaluation for VIA competencies during telemedicine mentorship using smartphone, Shiselweni, Eswatini, 2016–2018.

	Overall 6 months [95% CI]*	Smartphone-based Telemedicine Mentorship (1^st^ 3 months) [95% CI]	Smartphone-based Telemedicine Mentorship (2^nd^ 3 months) [95%CI]
**Agreement on Positive Cases (Sensitivity)**	100% [79·4%–100%]	100% [47·8%–100%]	100% [71·5%–100%]
**Agreement on Negative Cases (Specificity)**	95·7% [92·2%–97·9%]	95·9% [90·8%–98·7%]	95·4% [89·5%–98·5%]
AUC ROC*	0·978 [0·965 – 0·992]	0·98 [0·962–0·997]	0·977 [0·957 – 0·997]
PPV*	63·5% [48·7%–76·1%]	64·9% [44%–81·4%]	61·9% [40·9%–79·3%]
NPV*	100% [·%–·%]	100% [·%–·%]	100% [·%–·%]
**Cohen Kappa**	0·74 [0·58 – 0·89]	0·64 [0·36 – 0·93]	0·79 [0·61 – 0·96]

Among 247 patients who underwent smartphone-based VIA screening in 2018, 128 (49%) were HIV–positive; the mean age was 30.80 years (standard deviation [SD]: 7.74 years); 25/247 (10%) had previous VIA, of which 7/25 (28%) were positive, but only 3 received cryotherapy or loop electrosurgical excision procedure (LEEP); mean parity was 2.56 (SD: 1.2; range 0 to 9); and a history of sexually transmitted infections (STIs) was reported by 8/247 (3·2%) of women. There was a significant difference between VIA positivity in HIV–negative (4/129; 3.1%) versus HIV–positive (12/128; 10.2%) cases (*p* = 0.024). There was no significant difference between median age for HIV–negative (29 years old; interquartile range [IQR] 25 to 36) versus HIV–positive (34 years old; IQR 28 to 41) cases (*p* = 0·082). Logistic regression with age and HIV status in the model revealed that HIV–positive status (adjusted Odds Ratio [aOR]: 3.53, 95% confidence interval [CI]: 1.10 to 11.29; *p* = 0.033) and age (aOR: 1.06, 95% CI: 1.0004 to 1.13; *p* = 0.048) were conditionally associated with VIA positivity.

### Program-level VIA outcomes

**Fig 1:**
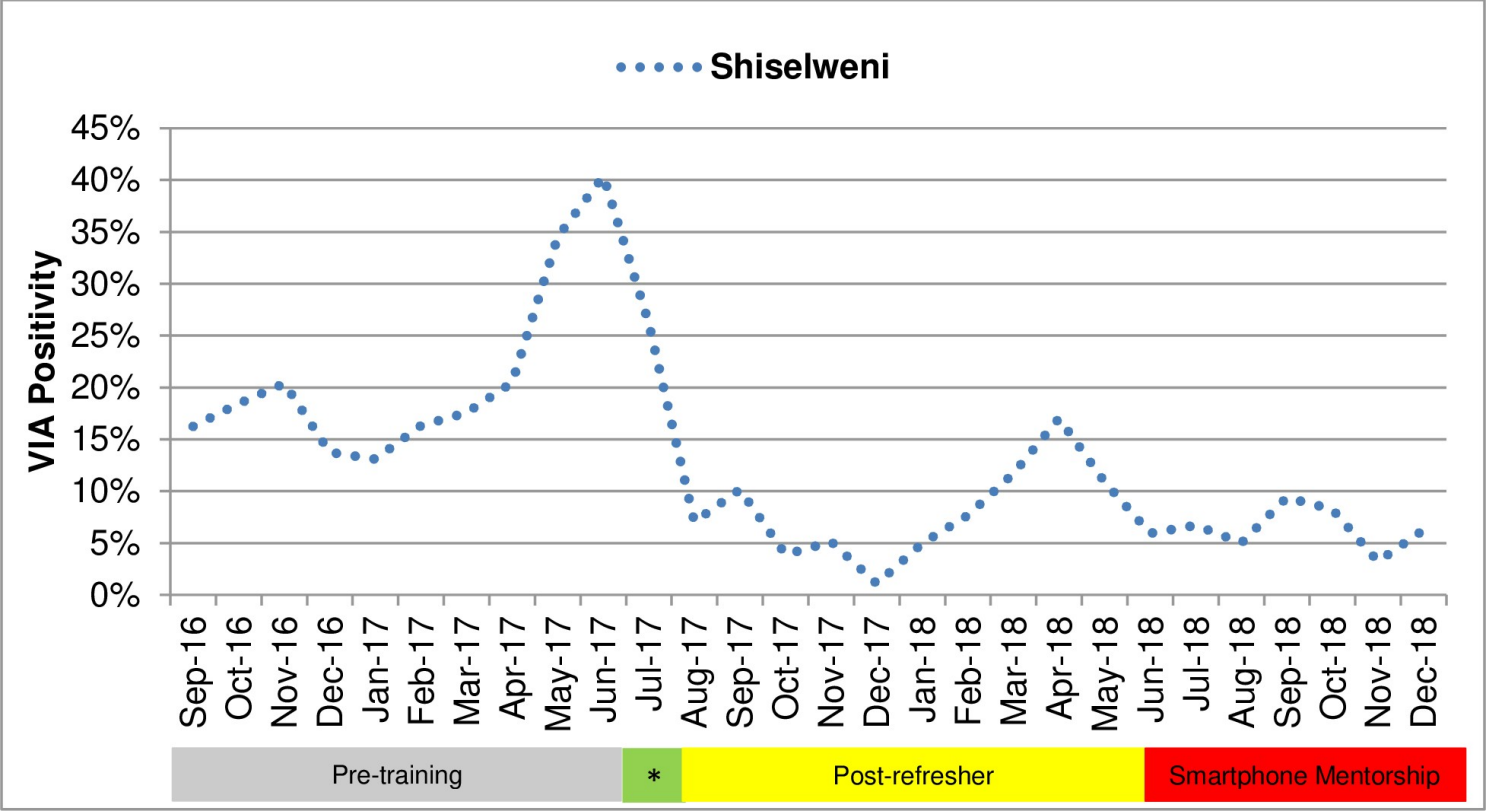
VIA positivity among patients undergoing VIA testing, Shiselweni, Eswatini, 2016–2018. *Refresher training. VIA, visual inspection of the cervix with acetic acid.

Overall, 4,247 patients underwent VIA screening and were included in this analysis. In 2016, after the initial VIA training, VIA positivity rate was 16%, which peaked at 40% right before the refresher training in July 2017. During the first half of 2017, before the refresher training, the average positivity rate was 25.1% (with a low of 14% in January 2017), which dropped to an average of 9.7% after the refresher training in the second half of 2017 (OR: 0.322; 95% CI: 0.259 to 0.400; *p* = 0.001). The positivity rate for second half of 2017 and first half of 2018 (after refresher) further dropped from 9.6% to 6.3% (OR: 0.976; 95% CI: 0.743 to 1.280; *p* = 0.959). Mentorship started in the second half of 2018 and helped maintain the low positivity rate around 6.3% on average. Overall positivity rate dropped from 16.5% in 2017 (which included post-refresher training phase) to 8.3% in 2018 (OR: 0.458; 95% CI: 0.374 to 0.562; *p* = 0.019), which was much closer to the expected level. Table 4 presents data and statistical analysis on positivity rates before and after the refresher training and the mentorship. Fig 1 presents a graph of VIA positivity during the study period.

**Table 4:** VIA positivity rates before and after refresher training (Pre- and Post-RefTrain) and before and after telemedicine smartphone-based VIA mentorship (Pre- and Post-Mentor), Shiselweni, Eswatini, 2016–2018.

Variable	Total n	VIA positive N (average %)	P value	Odds Ratio	95% Confidence interval
**Year 2017** vs.* Year 2018	2637 1610	436 (16·5) 134 (8·3)	0·019	0·458	[0·374-0·562]
**1**^**ST**^ **half 2017** (*Pre-RefTrain*)* **vs.** **2**^**nd**^ **half 2017** (Post-RefTrain)*	1168 1469	293 (25·1) 143 (9·7)	0·001	0·322	[0·259–0·400]
**2**^**nd**^ **half 2017** *(Post-RefTrain)* **vs.** **1st half 2018** *(Post-RefTrain)*	1469 1019	143 (9·7) 97(9·5)	0·959	0·976	[0·743-1·280]
**2**^**nd**^ **half 2017** *(Post-RefTrain)* **vs.** **2**^**nd**^ **half 2018** (Post-Mentor)*	1469 591	143 (9·7) 37 (6·3)	0·520	0·619	[0·426-0·901]
**2**^**nd**^ **half 2017 + 1**^**st**^ **half 2018** *(Post-RefTrain)* **vs.** **2**^**nd**^ **half 2018** *(Post-Mentor)*	2488 591	240(9·6) 37 (6·3)	0·517	0·625	[0·437-0·895]

**Fig 2:**
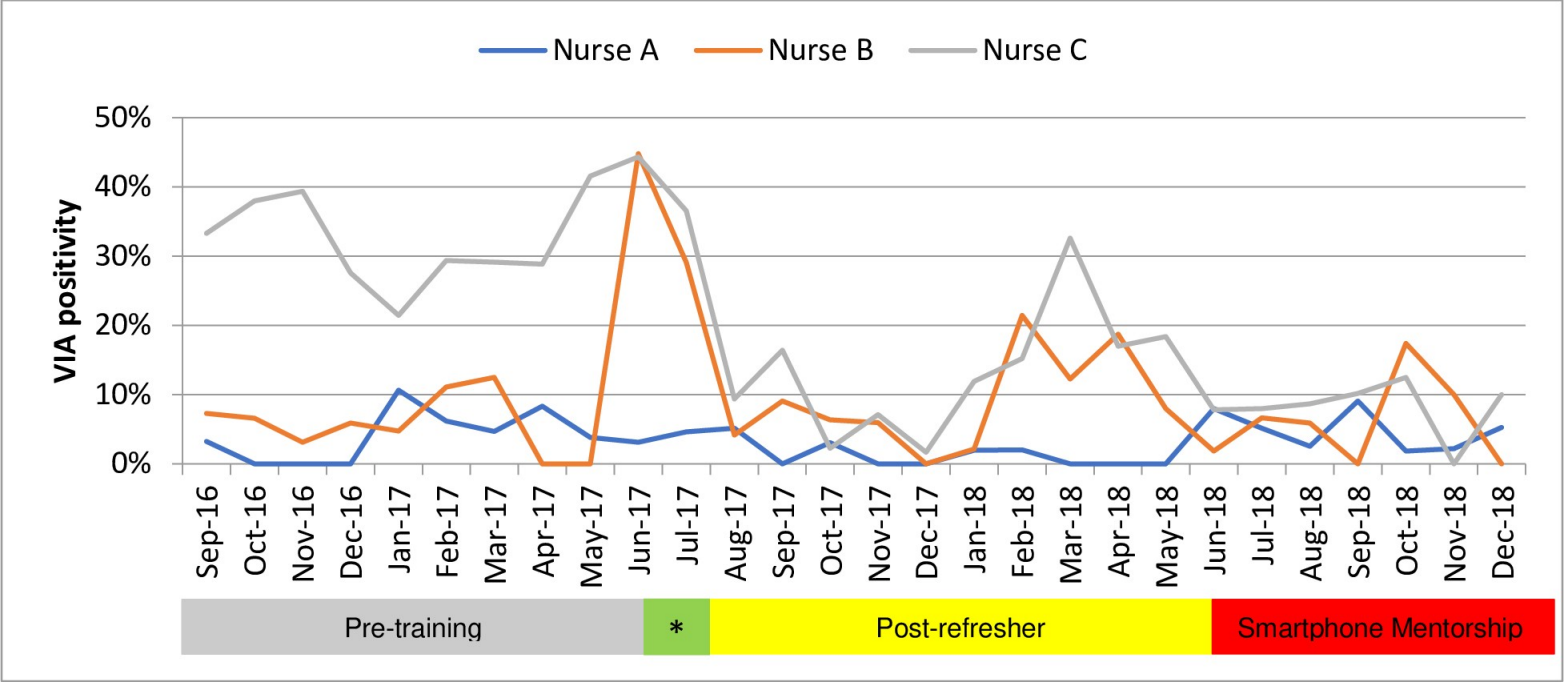
VIA positivity among patients undergoing VIA testing for individual nurses, Shiselweni, Eswatini, 2016–2018. *Refresher training. VIA, visual inspection of the cervix with acetic acid.

Nurses performed different numbers of VIAs, and there were individual differences over the 24-month period due to vacations, sick leaves, and other logistical constraints (Fig 2).

Changes in positivity rates over the 24-month period were statistically significant with 0.0076 percentage point decrease per month (Adj R^2^: 0.27, coefficient: −0.0076; standard error [SE]: 0.0024; CI: −0.0128 to −0.0025; *p* = 0.005).

### VIA treatment and cancer suspect

Treatment data for cryotherapy are only available for the year 2018. Among all patients receiving VIA in 2018 (*n* = 1,610), 135 (8.3%) were VIA–positive, of whom 119 (88%) were eligible for cryotherapy and 81 (68%) received cryotherapy the same day. The main reasons for non-eligibility were large and/or extending lesions inside the os or to the vaginal wall. These patients (*n* = 12) were referred to physicians. The main reasons for patients not receiving same-day cryotherapy were need for partner’s consent and temporary logistical problems with cryotherapy device (e.g., broken or needing maintenance), which were addressed. Due to unforeseeable logistical challenges in data collection methods and the MoH registry, data on cancer suspect cases are only available from September 2016 to February 2017 (*n* = 6; median age: 36 years) and from February 2018 to December 2018 (*n* = 4; median age: 45 years). Of 10 total cancer suspect cases, 5 (50%) were HIV–positive, and 5 (50%) were confirmed cancer by biopsy, of which 4 (80%) were HIV–positive.

As part of program evaluation, team meetings were frequently and consistently held to discuss program challenges and opportunities and elicited feedback from VIA providers, MoH managers, local and national health policy makers, patient population, and other stakeholders such as community informants and leaders. We incorporated feedback to adjust to logistical, sociocultural, and systems challenges.

## Discussion

Implementing VIA programs is recommended by WHO for cervical cancer screening in resource-limited settings as countless studies over the past 20 years have demonstrated acceptable test characteristics of VIA compared with cytology and colposcopy [2,4–8,14,15]. We developed and implemented an integrated mentorship training of VIA/digital cervicography via telemedicine using smartphones to improve VIA providers’ diagnostic and management competencies and skills retention. Our findings indicate that nurses significantly improved in VIA performance and maintained diagnostic competencies with acceptable agreement rates and Kappa statistic over time after the start of the mentorship. Gradual improvement in Cohen Kappa numbers with a substantial Kappa of 0.79 at the end of the 6-month mentorship are good indicators of the success of the training with reducing false VIA results. A Kappa of 0.64 at the end of the 3-month mentorship may suggest that mentorship duration could be shortened to accommodate logistical constraints if needed. We documented high agreement rates on positive and negative cases, high NPV, and high AUC, which provides more confidence in the VIA screening program in a screening-naïve population compared with other studies [14,15]. A modest PPV indicates the need for further improvement in decreasing false-positive cases. In such resource-poor settings, these are important findings as they ideally reduce the unnecessary treatment burden and the psychological impact and patient-level burden of false-positive and negative diagnoses.

As high false-positive rate was an important challenge, the program-level VIA positivity rate of 6.3%, much closer to expected level compared to that of the period before the training and mentorship, demonstrates the improved reliability and reproducibility of VIA screening in real-world settings. It is possible that there was a backlog of cases at the start of the program in the screening naïve population; however, the trend of increasing positivity rates over time and dramatic declines post-refresher and smartphone mentorship likely indicate the impact of training. Considering the actual burden of cervical cancer in Eswatini [11,23], the original VIA positivity rate of 16% immediately after the initial on-site training was high, an indication that short-exposure trainings are often inadequate in real-world settings. Even this relatively high positivity rate increased gradually after the initial training, consistent with the experience of attrition of VIA diagnostic competencies without more comprehensive or frequent training. In our program, the positivity rate improved significantly with the first refresher training; however, the impact of short-term refresher training also decreased over time evidenced by subsequent increasing positivity rates. This is likely due to the short intervention period during 1-time trainings that often lack adequate one-on-one teaching and provide an inadequate learning environment for adults [25,26]. After the start of the 6-month mentorship, the program-level data indicate improved diagnostic consistency and more realistic VIA positivity rates over time. These findings are especially important in the humanitarian and real-world setting of this project in contrast with other studies that have produced data in controlled settings [14,15]. We hypothesize that untreated HIV–positive status, selection bias in referral centers, providers’ inadequate competencies, lack of population level data, or lack of quality assurance processes may have contributed to higher positivity rates in other studies [11,14].

The success and impact of our intervention, we hypothesize, is related to the use of smartphones to facilitate VIA training and provide additional quality assurance. Our intervention applied components of Adult Learning Theory [25,26], including direct one-on-one feedback on nurses’ actual patients, explanation of reasoning, integration in the context of common tasks, self-direction to discover solutions, and experiential learning. We developed and implemented a process in which nurses learned from their peers during routine discussions of difficult cases and shared learning to address logistical barriers. Equally important, we hypothesize, the program functioned as quality assurance, thereby reinforcing adherence to diagnostic criteria. We were also able to provide direct, same-day, and on-site cryotherapy for more than two-thirds of eligible patients. Patients received their follow-up care immediately in a comfortable setting with a familiar provider. This approach decreased the burden on tertiary centers. Some patients could not receive treatment the same day, largely due to sociocultural issues. However, because the providers resided in the same communities, the likelihood of appropriate follow-up significantly improved, and most patients received cryotherapy within 3 to 6 months, which highlights the importance of decentralized approach and system strengthening at the primary care level.

Our data from HIV status and VIA positivity rates are consistent with other studies [11,15,27,29]. Data regarding cancer suspect cases are consistent across HIV status and age group and indicates that 50% of clinically cancer suspect cases are biopsy-proven cases. However, it may still be high and not generalizable as the screening was started in a screening-naïve population.

### Policy implications

There are important potential policy implications for smartphone-based VIA screening. Such successful program-level data could provide the impetus for large-scale task shifting with good-quality VIA. This is important because even with new advances with HPV testing, a reliable, good-quality, and functioning VIA or cytology program is required [2]. Many sub-Saharan countries have already implemented a healthcare system in which community health workers and nurses deliver primary care. With inexpensive smartphones equipped with quality digital cameras, cervicography no longer requires complex and expensive equipment [15–17,22]. To improve the sustainability, transportability, and scalability of the intervention effect, the program nurses served as trainers and provided mentorship to MoH nurses in a total of 30 facilities. The fact that the Shiselweni region is among the most remote and underserved areas in Eswatini, where there is limited access to healthcare and relatively inaccessible terrain, makes the scalability and transportability of this intervention to other similar settings more feasible.

This strategy is well suited for poor-resource settings and presents a unique opportunity for task shifting if training can be strengthened with innovative long-term strategies.

### Strengths and limitations

Our smartphone-based intervention was innovative for several reasons. First, trained nurses, who already had rapport with their communities and patients, delivered the intervention, possibly improving the acceptability, generalizability, and sustainability of the program, in contrast with episodic mass screening campaigns that often are not adequately adjusted to sociocultural contexts or health expectations and literacy. Mass campaigns also frequently do not provide repeat screening, which reduces the impact on incidence and mortality at the population level. Second, the implementation was in partnership with the MoH and its policy of VIA screening at the primary healthcare level, which may enhance sustainability and scalability. Third, the program relied on mobile phones that are ubiquitous in most African countries [28] and are used in health settings [29,30]. We did not observe any significant logistical issues regarding malfunctioning of the phones, internet, cellphone plans, electricity, or other related information technology (IT)-related challenges. Additionally, smartphone-based training and mentorship is less costly and more accessible than traditional quality assurance approaches or refreshers.

We did not use any cytology or colposcopy to assess diagnostic validity of VIA images; however, extensive studies over the past decades have demonstrated comparable VIA test characteristics with those of cytology and colposcopy, and other studies have also demonstrated validity of expert agreement for assessing quality of VIA diagnosis. During the project implementation, we faced some logistical challenges regarding the resources needed to collect all relevant program-level data, documentation of patient-level indicators, logistics of cryotherapy device maintenance, consistent gas supplies, and infection control materials. However, these challenges did not impede the overall implementation of the program. The most important data related to outcomes were consistently and accurately collected. Conducting a program evaluation in a real-world and humanitarian context, we were not able to implement any control group. Therefore, there are potential biases in our single-arm quasi-experimental design in this setting. However, we hypothesize that our different training interventions and temporal relationship with trends in program-level data over time provide an acceptable level of evidence.

## Conclusions

Our findings suggest overall efficacy, feasibility, and potential effectiveness of VIA training and mentorship using smartphone imaging for cervical cancer screening. They also provide important information on approaches to improve reliability and reproducibility of VIA diagnostics outside study settings. The VIA training and mentorship were built based on the elements of Adult Learning Theory to provide an experiential learning environment and foster intellectual contribution. The use of smartphone imaging provided additional benefits such as peer-to-peer education, remote quality services to hard-to-reach populations, and increased adherence to quality control services. Effective collaborations, manageable logistical challenges, decreased burden on tertiary centers, VIA services in familiar and sustainable settings, improved local capacity building, and quality and affordable services were important achievements. Overall, the program shows promise for improving the effectiveness of VIA screening and the reliability, reproducibility, scalability, and quality of VIA programs to address the cervical cancer burden in resource-poor settings.

## Supporting information

S1 STROBE Checklist Strengthening the Reporting of Observational Studies in Epidemiology (STROBE) guideline. (DOCX)

S1 CaseTemplate Template to submit case information to reviewer. (DOCX)

S1 Text Instruction to perform quality cervical imaging. (DOCX)

## Abbreviations:

aOR: adjusted Odds Ratio
AUC ROC: area under the curve of receiver operating characteristics
CI: confidence interval
HPV: human papillomavirus
IQR: interquartile range
IT: information technology
JHPIEGO: Johns Hopkins Program for International Education in Gynecology and Obstetrics
LEEP: loop electrosurgical excision procedure
MoH: Ministry of Health
MSF: Medecins Sans Frontieres
NPV: negative predictive value
OR: odds ratio
PHI: protected health information
PPV: positive predictive value
SD: standard deviation
SE: standard error
STI: sexually transmitted infection
STROBE: Strengthening the Reporting of Observational Studies in Epidemiology
TB: tuberculosis
VIA: visual inspection of the cervix with acetic acid
WHO: World Health Organization
