# Supplementary material for: Evaluating smartphone strategies for reliability, reproducibility, and quality of VIA for cervical cancer screening in the Shiselweni region of Eswatini: A cohort study
Source: PLoS Med. 2020 Nov 19;17(11):e1003378. doi: 10.1371/journal.pmed.1003378 (PMC7676712; doi:10.1371/journal.pmed.1003378)
Supplement: S1 CaseTemplate — (DOCX) [file pmed.1003378.s002.docx]

**S1 CaseTemplate**: Template to Submit Case Information to Reviewer

| **Date** | **Register Code #** | **Age** | **HIV Status** | **Previous screening** | **Previous Cryo** | **Diagnosis** | | | | **Notes** | **Parity** | **STIs** | **Cervix Status (normal vs abnormal)** | **Lib*** | **Mentor's comments** | **Cervical Images** |
| --- | --- | --- | --- | --- | --- | --- | --- | --- | --- | --- | --- | --- | --- | --- | --- | --- |
|  |  |  |  |  |  | **Pos** | **Neg** | **Post Cryo** | **> 75% lesion** |  |  |  |  |  |  |  |
|  |  |  |  |  |  |  |  |  |  |  |  |  |  |  |  |  |
|  |  |  |  |  |  |  |  |  |  |  |  |  |  |  |  |  |
|  |  |  |  |  |  |  |  |  |  |  |  |  |  |  |  |  |
|  |  |  |  |  |  |  |  |  |  |  |  |  |  |  |  |  |
|  |  |  |  |  |  |  |  |  |  |  |  |  |  |  |  |  |
|  |  |  |  |  |  |  |  |  |  |  |  |  |  |  |  |  |
|  |  |  |  |  |  |  |  |  |  |  |  |  |  |  |  |  |
|  |  |  |  |  |  |  |  |  |  |  |  |  |  |  |  |  |
|  |  |  |  |  |  |  |  |  |  |  |  |  |  |  |  |  |
|  |  |  |  |  |  |  |  |  |  |  |  |  |  |  |  |  |
|  |  |  |  |  |  |  |  |  |  |  |  |  |  |  |  |  |
|  |  |  |  |  |  |  |  |  |  |  |  |  |  |  |  |  |

*Lib – for library, refers to images that are suitable for training purposes
