# Supplementary material for: Evaluating smartphone strategies for reliability, reproducibility, and quality of VIA for cervical cancer screening in the Shiselweni region of Eswatini: A cohort study
Source: PLoS Med. 2020 Nov 19;17(11):e1003378. doi: 10.1371/journal.pmed.1003378 (PMC7676712; doi:10.1371/journal.pmed.1003378)
Supplement: S1 Text — (DOCX) [file pmed.1003378.s003.docx]

**S1 Text**: Instruction to Perform Quality Cervical Imaging

- Consent comes first. Describe the procedure. Confidentiality. Make sure they understand no part of their identifiable body parts or personal information is recorded with the photos.
  - Patient often like to see the image of their own cervix which could be a good opportunity to do health education and STI prevention too
- Use standing lamp to zoom light on overall Vulva/Genital area and avoid direct light focused on the cervix
- Use disposable speculum if you can to minimize light reflection. If not available, you need to change your camera angle depending on patient anatomy and position to avoid light reflection on the metal speculum
- Use digital camera with its flash on. Get close to vaginal opening as much as you can. Avoid auto-focus and instead manually focus camera to take a photo using camera’s flash
- Change your angle if needed and take more images, and finally pick the one that has the best clarity and the least light reflection
  - It comes to you with more practice!
